# Breastfeeding assistance for preterm and low birth weight infants: best practices implementation project*

**DOI:** 10.1590/1980-220X-reeusp-2023-0380en

**Published:** 2024-06-28

**Authors:** Camila Medeiros Cruvinel Cunha, Eliane de Fátima Almeida Lima, Dulce Maria Pereira Garcia Galvão, Ana Paula Almeida Brito, Luciana Mara Monti Fonseca, Cândida Caniçali Primo

**Affiliations:** 1 Universidade Federal do Espírito Santo, Centro de Ciências da Saúde, Departamento de Enfermagem, Vitória, ES, Brazil.; 2 Escola Superior de Enfermagem de Coimbra, UCP Enfermagem de Saúde da Criança e do Adolescente, Unidade de Investigação em Ciências da Saúde: Enfermagem, Coimbra, Portugal.; 3 Universidade de São Paulo, Hospital Universitário, São Paulo, SP, Brazil.; 4 Centro Brasileiro para o Cuidado à Saúde Baseada em Evidência: Centro de Excelência do JBI, São Paulo, SP, Brazil.; 5 Universidade de São Paulo, Escola de Enfermagem de Ribeirão Preto, Ribeirão Preto, SP, Brazil.

**Keywords:** Breast Feeding, Infant, Premature, Infant, Low Birth Weight, Neonatal Nursing, Implementation Science, Lactancia Materna, Recien Nacido Prematuro, Recién Nacido de Bajo Peso, Enfermería Neonatal, Ciencia de la Implementación., Aleitamento Materno, Recém-Nascido Prematuro, Recém-nascido de Baixo Peso, Enfermagem Neonatal, Ciência da Implementação

## Abstract

**Objective:**

To describe the process of best practices implementation for breastfeeding assistance for preterm and low birth weight infants.

**Method:**

Participatory research that used the evidence implementation methodology of the JBI, held at a university hospital in southeastern Brazil, with the participation of a multidisciplinary team and managers.

**Stages:**

Situational diagnosis, baseline audit and feedback, protocol development, training, implementation, and monitoring.

**Results:**

Seven audit criteria were defined. In the baseline audit, three criteria were met, with eleven barriers to be resolved being listed. The strategies carried out were protocol development and multidisciplinary and intersectoral training. After the training, compliance was achieved with the seven criteria audited in the first follow-up audit and five in the second, emphasizing the increase in compliance after the implementation of the outlined strategies.

**Conclusion:**

The project achieved the objective of improving evidence-based practice, and allowed the implementation of the institution's first breastfeeding protocol. However, it shows the need to maintain training for adherence and enculturation of new practices.

## RESUMO

**Objetivo:**

Descrever o processo de implantação das melhores práticas para assistência à amamentação de recém-nascido prematuro e de baixo peso.

**Método:**

Pesquisa participativa que utilizou a metodologia de implementação de evidências do JBI, realizada em hospital universitário no sudeste do Brasil, com participação de equipe multiprofissional e gestores.

**Etapas:**

diagnóstico situacional, auditoria de base e feedback, elaboração de protocolo, treinamentos, implementação e monitoramento.

**Resultados:**

Foram definidos sete critérios de auditoria. Na auditoria de base, três critérios se mostraram conformes, sendo elencadas onze barreiras para serem solucionadas. As estratégias realizadas foram elaboração de protocolo e treinamentos multidisciplinares e intersetoriais. Após as capacitações obteve-se conformidade dos sete critérios auditados na primeira auditoria de seguimento e de cinco na segunda, enfatizando o aumento da conformidade após concretização das estratégias traçadas.

**Conclusão:**

O projeto alcançou o objetivo de melhorar a prática baseada em evidências, e possibilitou a implementação do primeiro protocolo de amamentação da instituição, porém evidencia a necessidade de manutenção dos treinamentos para adesão e enculturação das novas práticas.

## RESUMEN

**Objetivo:**

Describir el proceso de implantación de las mejores prácticas para la asistencia a la lactancia materna en recién nacidos prematuros y de bajo peso al nacer.

**Método:**

Investigación participativa que utilizó la metodología de implementación de evidencia del JBI, realizado en un hospital universitario del sudeste de Brasil, con la participación de un equipo multidisciplinario y gestores.

**Etapas:**

Diagnóstico situacional, auditoría de base y retroalimentación, desarrollo de protocolo, capacitación, implementación y seguimiento.

**Resultados:**

Se definieron siete criterios de auditoría. En la auditoría de base se cumplieron tres criterios y se enumeraron once barreras por resolver. Las estrategias implementadas fueron el desarrollo de protocolo y la capacitación multidisciplinaria e intersectorial. Luego de la capacitación se logró el cumplimiento de los siete criterios auditados en la primera auditoría de seguimiento y de cinco en la segunda, destacando el incremento en el cumplimiento luego de implementar las estrategias trazadas.

**Conclusión:**

El proyecto logró el objetivo de mejorar la práctica basada en evidencia y permitió la implementación del primer protocolo de lactancia materna de la institución, pero destaca la necesidad de mantener la capacitación para la adherencia y la inculturación de nuevas prácticas.

## INTRODUCTION

Preterm birth is an important risk factor for infant morbidity and mortality. Approximately 60 to 80% of infants who die are preterm and/or small for gestational age. Preterm babies have a gestational age below 37 weeks at birth and low birth weight infants weigh less than 2.5 kg. Preterm and low birth weight infants have a 2 to 10 times higher risk of mortality than full-term infants with normal birth weight^(1,2)^.

Preterm infants present anatomophysiological immaturities that interfere with breastfeeding, reflected by the difficulty in latching and maintaining it on the mother’s breast, and by the lack of strength to extract milk during breastfeeding^(3,4)^. Furthermore, breastfeeding in preterm and low birth weight infants can be made difficult by prolonged hospitalization and challenges during the infant’s stay in the Neonatal Unit, such as: mother-baby separation, delay in enteral and oral feeding, maternal illness and stress, maternal care behavior, insufficient knowledge and skills of health professionals, and the need for logistical support for the mother to breastfeed^(5–7)^.

Breastfeeding is very important for preterm infants (PTI), as it helps with gastrointestinal maturation, prevents cases of infection and early sepsis, reduces the rates of necrotizing enterocolitis and retinopathy of prematurity, helps in the mother-child bond and in the best neurobehavioral performance, also reducing hospital readmissions^(2,3,6,8)^. It should be noted, however, that breastfeeding is a complex, dynamic process and depends on variables that can have positive or negative influence^(9,10)^.

According to the recommendations of the World Health Organization, based on the best scientific evidence, the establishment and maintenance of breastfeeding in preterm and low birth weight infants require health professionals to have knowledge and skills on lactation and provide breastfeeding support, including provision of specific prenatal information and care. Early, continuous, and prolonged skin-to-skin contact (kangaroo mother care), early initiation of breastfeeding, and mothers’ access to guidance and support for breastfeeding throughout the baby’s hospitalization are decisive actions for the establishment of exclusive breastfeeding for this population^(2,7,11,12)^.

Preterm infants breastfeeding has some particularities that complicate the process, making early weaning a common factor in this setting^(13)^. Numerous studies address the low rates of exclusive breastfeeding of preterm and low birth weight newborns at hospital discharge, evidenced by factors ranging from the delay in direct suckling of the mother’s breast, the distance between mother and newborn from the first minutes of life, to the difficulties of maintaining the mother’s milk production and the lack of support in hospitals and at home^(9,13)^. Some authors point out that the infant’s first oral feeding method, as well as the mother’s level of education and her attachment to the newborn, are critical predictors of the success of breastfeeding in preterm infants. Mothers should be encouraged to breastfeed on their baby’s first oral attempt and strategic breastfeeding support measures should be provided before oral feeding is started^(6,9)^.

In this scenario, the importance and challenge for health professionals of implementing interventions based on the best scientific evidence stand out, and the JBI mission is to facilitate the implementation of the best evidence available in healthcare. JBI is an international research and development organization, specialized in providing healthcare professionals with resources for clinical practice based on scientific evidence^(14,15)^.

Although the recommendations are available in the literature, the hospital where the research was carried out had not yet implemented a protocol based on scientific evidence for breastfeeding assistance for preterm and low birth weight infants, thus resulting in different behaviors from the professionals. In this regard, this study aims to describe the process of best practices implementation for breastfeeding assistance for preterm and low birth weight infants.

## METHOD

### Design of Study

Participatory study using the theoretical framework of the Evidence-Based Health Care Model (*CSBE*)^(16)^ and the JBI Evidence Implementation Methodology^(17)^. The JBI implementation approach is grounded in the audit and feedback process, along with a structured approach to identifying and managing barriers and facilitators to compliance with recommended clinical practices^(15)^.

### Location, Study Population, Selection Criteria and Sample Definition

The research was carried out in the Neonatal Unit (NU) of a university hospital in the state of Espírito Santo, Brazil, belonging to the hospital network of the Brazilian Hospital Services Company (*EBSERH*), with care entirely focused on users of the Brazilian Public Health System (*SUS*), not accredited with the Baby-Friendly Hospital Initiative (BFHI), and state reference in high-risk pregnancies. The NU has 23 beds divided among the Neonatal Intensive Care Unit (NICU) with 10 beds, the Conventional Intermediate Care Unit (CoIMCU) with 10 beds, and the Kangaroo Intermediate Care Unit (KIMCU) with 3 beds.

The working group in charge of the research was created in May 2022, through the invitation of professionals from different categories, all with direct or indirect involvement in breastfeeding. The number of participants in the group varied over the months, however there were the guarantee of always having people from different categories and sectors so that resolutions could be agreed in an interdisciplinary and intersectoral manner. The invitation to participate in the group was made directly to managers and representatives of each professional class who have historically been more engaged with preterm and low birth infants breastfeeding in the service.

Although participation was voluntary, all meetings were attended by professionals from the neonatal unit, human milk bank, and maternity ward. Meeting dates were announced a month in advance and confirmed again a week before. If someone gave up, a new member of the same professional class was recruited, considering the degree of interest shown by the employee in the topic and the scope of the different work shifts. Examples of actors in this process are: technical managers for each service, representatives of nursing, medicine, and speech therapy (on a fixed basis), and nursing technicians, social workers, occupational therapists, and psychologists (on a floating basis). The classes with the greatest participation in the development of the protocol were: nursing, medicine, and speech therapy. The number of participants in each meeting was around eight to ten professionals. There was no calculation for the minimum number of participants.

For the situational diagnosis of breastfeeding routines in the sector and better understanding of which points deserved greater attention during the protocol development, the working group chose to promote the participation of employees and mothers of preterm and low birth weight infants of the neonatal unit, through questionnaires and interviews, respectively.

The number of employees was random, but we sought to reach a minimum of 50% of the neonatal unit team. Coincidentally, there was the same number of employees participating in the baseline audits and the first follow-up audit, with a total of 62 professionals (47% of the team), and greater participation in the second follow-up audit, in which 73 professionals participated (55% of the neonatal unit team). It is not possible to say that the participants were the same in all stages of the research, but it is possible to observe a tendency for the same people to engage with the topic.

The number of mothers was defined as per guidance in the BFHI manual, which requests a sample of at least 10 mothers for the quantity worked. The sample involved mothers of babies admitted to the neonatal unit more than 6 hours after birth, who visited their children and agreed to voluntarily respond to the interviews, carried out exclusively by the person responsible for the research and based on a specific questionnaire from the BFHI Manual. This sample was obtained in the baseline and first follow-up audits, being surpassed in the second follow-up audit, which included the participation of 12 mothers.

### Data Collection

In May 2022, the first meeting of the working group was held, in person, with managers and representatives of the main categories working in the neonatal unit, to design the project and identify the audit criteria based on the best scientific evidence and methods to measure compliance with best practices. The criteria were based on evidence summaries from the JBI on preterm and low birth weight infants breastfeeding and in the BFHI Training Course Guide for maternity teams from the World Health Organization. The best practices related to preterm and low birth weight infants breastfeeding selected were^(2,8,11,12,18)^:

- Mothers of preterm infants who wish to feed their babies with human milk should begin expressing milk as soon as possible after the baby is born, for a minimum of eight times a day, and up to 12 times during the first few weeks when lactation is being established (Grade B evidence).
- Health professionals should educate mothers about the benefits of breastfeeding, ideal timing and frequency of expressing milk, and strategies or techniques for effective expression of human milk (e.g., breast massage and skin-to-skin contact). These guidelines should be given during pregnancy and emphasized again when preterm birth occurs (Grade B evidence).
- Kangaroo mother care is recommended as a strategy to increase the rate of breastfeeding in low birth weight and very low birth weight infants (Grade A evidence).
- Low birth weight infants who can be breastfed and are clinically stable should be placed on the breast as soon as possible after birth (Grade A evidence).
- Kangaroo care should be encouraged for preterm and low birth weight babies who are clinically stable, as long as it is viable and for as long as possible (Grade A evidence).
- Low birth weight babies should be exclusively breastfed until six months of age (Grade A evidence).
- The use of bottles and pacifiers should be avoided while the baby is learning to breastfeed. If necessary, alternative feeding methods, with expressed breast milk or supplemental formula, include cup and tube feeding (Grade B Evidence).

The audit criteria used in this project are described in Chart 1, as well as the sample and methods used to measure compliance with best practices.

**Chart 1:** Description of the evidence-based audit criteria used in the project (baseline and follow-up audits), the sample, and the approach to measuring compliance with best practices for each criteria audited – Vitória, ES, Brazil, 2023.

Audit criteria	Sample	Method used to check compliance with best practices
1. Healthcare professionals are trained in best practices	Professionals 62 employees in the baseline and follow-up audit	Individual questionnaire with open and closed questions applied to employees in the sector Question: Have you taken any course on Breastfeeding or received practical training in breastfeeding since joining the team? ( ) No ( ) Yes Considered compliant if the employee answers “yes”
2. Health professionals advise mothers on breastfeeding	Professionals 62 employees in the baseline and follow-up audit	Individual questionnaire with open and closed questions applied to employees in the sector Question: Do you feel safe to provide guidance related to the practice of breastfeeding for preterm and low birth weight infants? ( ) No ( ) Yes ( ) Partially Considered compliant if the employee answers “yes”
3. PTI are placed on the breast when clinically stable	Professionals 62 employees in the baseline and follow-up audit	Individual questionnaire with open and closed questions applied to employees in the sector Question: Do you assist in the breastfeeding of preterm and low birth weight newborns as soon as they become stable? ( ) No ( ) Yes ( ) Partially Considered compliant if the employee answers “yes”
4. Mothers of PTI who wish to breastfeed are advised to begin lactation and expressing milk as soon as possible after birth, if breastfeeding is not possible.	Mothers 10 mothers in the baseline and follow-up audit	Individual interview with the baby’s mother admitted to the Special Care Unit Question: Did anyone on the team offer to help you initiate and maintain lactation? ( ) No ( ) Yes Considered compliant if the mother answers “yes”
5. Mothers of PTI/LBW infants are advised on the benefits of breastfeeding, the frequency of milk expression and techniques for effective expression of milk	Mothers 10 mothers in the baseline and follow-up audit	Individual interview with the baby’s mother admitted to the Special Care Unit Questions: Did anyone on the team show you how to express breast milk by hand and inform how often this should be done to maintain lactation? ( ) No ( ) Yes Considered compliant if the mother answers “yes” or answers a number equal to or greater than 8x/day
6. Mothers of clinically stable PTI/LBW are encouraged to adopt the kangaroo care	Mothers 10 mothers in the baseline and follow-up audit	Individual interview with the baby’s mother admitted to the Special Care Unit Question: Did you have the opportunity to hold your baby in direct contact? ( ) No ( ) Yes If not, was there any justification? Considered compliant if the mother answers “yes” or explains the “no” answer.
7. Alternative feeding methods with expressed breast milk or supplemental formula, including tube and cup, are used safely	Professionals 62 employees in the baseline and follow-up audit	Individual questionnaire with open and closed questions applied to employees in the sector Question: Check the techniques you think you have the ability to perform: ( ) Breastfeeding assistance ( ) Translactation ( ) Gavage ( ) Cup ( ) Finger feeding Considered compliant if the employee selects one or more options. The result will show compliance if the sum of the techniques performed by the professionals reaches a percentage greater than 80%.

Note: PTI – Preterm infant; LBW – Low birth weight.

The baseline audit was carried out over 20 days, between June 10 and 30, 2022, using the Google Forms platform. For criteria 1, 2, 3 and 7, an online questionnaire was answered by NU employees with direct or indirect involvement in breastfeeding. Those who were not working in person at the unit during the study period were excluded from the research. Participants were: nursing, medicine, physiotherapy, speech therapy, psychology, social work, occupational therapy, secretarial, and hygiene services professionals. The questionnaires were sent online to all employees of the neonatal unit, with a specific computer available to answer them during a few afternoons, and assistance offered, provided by the person in charge of the research, to access Google Forms.

For criteria 4, 5 and 6, interviews were carried out with mothers of preterm or low birth weight infants in the unit, for convenience, at the bedside, after visiting the newborns. Mothers whose babies had been born less than 6 hours before and/or presented unstable status (mother or baby) were excluded from the interviews.

The questionnaire for health professionals and the instrument for interviewing mothers were established after the first working group meeting. The questionnaire was based on the audit criteria and based on Module 4 - Self-assessment and monitoring of BFHI Hospitals (with some modifications to adapt to the reality of the NU). The questionnaire consisted of two parts, one for characterizing the participants and the other for situational diagnosis, containing 18 open and closed questions that addressed the main aspects and knowledge of professionals on the topic.

For the interview with the mothers, a document available in Module 4 was used - *Self-assessment and monitoring of BFHI Hospitals - Instrument for monitoring Baby-Friendly Hospitals Part II: C - Interview with the mother of babies admitted to the Special Care Unit.* This instrument consisted of eight closed questions with the possibility for the mother to comment on each one.

The working group meetings took place monthly, in the months of September, October, November and December 2022. They lasted two hours and were coordinated by the NU clinical nurse, project leader, and participant in the institution’s Breastfeeding Committee, established in 2022.

The results of the baseline audit were presented to the working group in September. Throughout the remaining meetings, the aim was to: discuss the results of the baseline audit, identify barriers to implementing best practice recommendations, propose strategies to overcome these barriers, and determine the resources needed for successful implementation. Suggestions and observations from the components of the working group were made during the meetings and were sent via electronic message to enable adjustments in a more dynamic way.

The strategies were implemented through the creation of documents related to the topic (such as protocol, standard operating procedures, flowcharts, and process mapping), with theoretical-practical training and the beginning of the structuring of support groups in the service. Stakeholders were encouraged to participate in the planning established to improve clinical practice. The tool *Getting Research into Practice (GRiP)* from the JBI was used to document the barriers encountered, as well as the resources required and strategies implemented to improve compliance with the audited criteria.

The members of the nursing, medical, and speech therapy teams took on a prominent role, participating permanently in the working group and, subsequently, becoming a reference for disseminating best practices. All decisions and pacts established in the working group were replicated by these actors in the sector’s routine.

The aim is also to promote biannual follow-up audits and protocol updates every 2 years, according to the institution’s routine.

### Impact and Sustainability

The training of professionals took place through theoretical-practical workshops held in January and February 2023, formulated on best evidence-based practices, covering the following contents: Anatomy and physiology of lactation; Benefits of breastfeeding; Supportive practices (kangaroo mother care, skin-to-skin contact, and expressing milk at the bedside); BFHI and related policies; Guidance for professionals regarding the routine of mothers of preterm infants within the institution; Transition from tube to oral diet and demonstration of related techniques; Rights and duties of the family in a hospital environment; and Breastfeeding advice.

After training, professionals responded, individually, to the same questionnaire applied in the baseline audit to check adherence and implementation of best practices. The same baseline audit criteria, sample size, and audit methods were used in the first follow-up audit, carried out from March 4 to March 24, 2023. In the period from July 3 to 23, 2023, there was a second follow-up audit using the same instruments, also carried out for convenience, but with a larger sample of professionals and mothers. This increase took place spontaneously, probably due to greater participation of both in the process.

The data collected in the audits were entered into the *Practical Application of Clinical Evidence System (PACES)* from JBI.

The sustainability of the result will be supported by constant updating of the protocol, ongoing education, and the performance of new follow-up audits, scheduled to evaluate the change in behavior and ensure the accomplishment of the new practices.

### Data Analysis and Treatment

JBI’s PACES was used to analyze the data, which include automated reports of percentage changes on compliance with the audited criteria.

### Ethical Aspects

Participants were personally informed about the study and, after reading, signed the Free Informed Consent Form. They were also informed of their right to refuse participation, stop the interview or withdraw from the study at any time, without any impact on their future assistance/services. The research was approved by the Ethics and Research Committee in 2022 with opinion number 5.519.362 and is in accordance with Resolution 466/12.

## RESULTS

### Baseline Audit

At this stage, the research involved the participation of 62 employees and 10 mothers. It was identified that criteria 4, 5 and 7 had a high compliance with best practices (greater than 80%). The other criteria analyzed had low compliance, varying between 53.3% (criterion 1) and 74.2% (criterion 3). Criteria 2 and 6 reached 55.8 and 70% respectively.

The number of breastfeeding trainings offered by the hospital is low, totaling a percentage of 53.3% of employees who took a course in the area, characterizing low compliance for criterion 1.

For criterion 2, when employees were asked about their safety in providing guidance related to preterm and low birth weight infants breastfeeding, 55.8% of them said they felt safe. The remaining said they felt insecure or partially safe.

For criterion 3, 74.2% compliance was detected when the team was asked whether PTIs were placed on the breast as soon as they were clinically stable. It should be noted that this criterion represented the point of greatest conflict for the planning team during the preparation of the protocol.

For criterion 4, it was found that 100% of mothers reported being instructed on the importance of breastfeeding and the benefits of breast milk. The unit has a strong culture of valuing breast milk and the need to maintain mothers’ milk production, preserving the chances of having exclusive breastfeeding.

Criterion 5 was analyzed based on the responses of PTI/LBW’s mothers and investigated whether they were advised on breast stimulation, early and frequent milk expression to maintain milk production, and frequency of feedings. This audit criterion also achieved 100% compliance.

Criterium 6 addressed whether the mothers of clinically stable PTI/LBW are encouraged to adopt the kangaroo care. A total of 70% of mothers stated that they were guided and encouraged to carry out the method.

Finally, criterion 7 addressed the safety of employees to carry out alternative methods of feeding the newborn. A total of 83.88% of them said they felt safe performing methods such as: gavage, translactation, cup, and finger feeding. The way the questionnaire was prepared allowed the professional to select as many alternatives as he deemed necessary.

### Strategies for Putting Research into Practice (GRiP)

Based on the data collected in the baseline audit, the working group identified eleven barriers to complying with best practice recommendations; corresponding strategies and resources have been developed to address these barriers (listed in Chart 2).

**Chart 2:** Barriers found, strategies, resources, and results achieved – Vitória, ES, Brazil, 2023.

Barriers found	Strategies	Resources	Results
1. Lack of breastfeeding protocol for PTI/LBW infants; reduced number of documents on the topic in the institution	Preparation of protocol, SOPs, flowcharts, and process mappings	Multidisciplinary working group; Breastfeeding Commission	Working group established; Protocol and documents prepared and approved
2. Low supply of breastfeeding training in the hospital	Training of health professionals who serve the binomial	Auditorium; Multimedia; Didactic resources	Trained team
3. Maternal absence and lack of routine for PTI/LBW mothers within the institution	Preparation of process mapping; Holding of support groups with mothers	Auditorium; Multimedia; Serial album; Informative folders; Multidisciplinary and intersectoral working group	Creation and compliance with the role of employees in the routine of the mother of PTI/LBW; Preparation of invitations for employees interested in participating in permanent support groups
4. Professionals feel insecure or partially safe when providing guidance for establishing and maintaining breastfeeding	Training of professionals who serve the binomial; Develop and implement flowcharts related to breastfeeding	Classroom; Multimedia; Didactic resources; Serial album	Mothers guided daily by professionals; Implementation of the Flowchart for Referring Mothers to the HMB
5. Divergence in professional conduct and guidance	Preparation of the protocol; Training of professionals	Multidisciplinary working group; Classroom; Multimedia; Didactic resources	Protocol drawn up and approved; Trained team and uniformly oriented mothers
6. Discontinuity in the work carried out between the sectors in which PTI/LBW breastfeeding occurs	Training of professionals; Development of an instrument for dialogue between sectors that serve the binomial	Binomial characterization instrument	Team in the training process; Intersectoral communication instrument in the validation phase in the hospital
7. Mothers have difficulty performing stimulation, milk expression, and resistance to placing PTNB/LBW infants in kangaroo position	Conversation circles and individual guidance	Ward/Unit/Auditorium; Serial album; Information folder; Cartoon; Didactic resources	All mothers perform lactation maintenance and kangaroo position (when the newborn is stable)
8. Difficulty establishing when to begin the tube-to-oral transition	Seminar: Breastfeeding preterm and low birth weight infants: transition from tube to oral diet	Classroom; Multimedia; Didactic resources	Greater dialogue between professionals, systematic and individualized reassessments, team training in best evidence-based practices
9. Lack of knowledge of the Ten Steps to Successful Breastfeeding as guidelines for breastfeeding in the institution	BFHI Trainings	Auditorium; Multimedia; Dynamics	Knowledge and reproduction of the Ten Steps to Successful Breastfeeding in the work routine
10. Lack of collection site at the institution	Conversations with the institution’s management board	Location and own team	Ensure a place that operates 24 hours a day to extract milk and maintain mothers’ milk production
11. Offering supplements, bottles and pacifiers for various reasons without documents supporting their use	Preparation of documents	Multidisciplinary working group; Breastfeeding Commission	Regulate the use of the complement and devices only in special situations; Documents in the preparation phase

Note: PTI - Preterm infant; LBW - Low birth weight.

Firstly, there was a reduced number of protocols and documents on breastfeeding in the institution, especially for premature babies. As a strategy, a multidisciplinary working group and breastfeeding committee were established to produce and implement established routines, through documents such as: Breastfeeding and Woman-Friendly Care Policy; Breastfeeding Protocol for preterm and low birth weight infants; Standard Operating Procedures (Maintenance of milk production of nursing mothers with babies admitted to the NICU, breast massage, manual breast milking technique at the bedside, and hygiene and behavior of milk donors); Flowchart for referring PTI/LBW mothers to the HMB and process mapping regarding the embracement of PTI/LBW mothers at the institution.

Secondly, there was a low supply of courses on the subject in the hospital. As a strategy, theoretical-practical training was carried out for employees of the neonatal unit in conjunction with other sectors where breastfeeding services to the binomial are provided.

Thirdly, maternal absence was highlighted as one of the main challenges in the breastfeeding process. As a resource, process mapping was created with employees’ responsibilities in the routine of PTI/LBW mothers in the hospital, in addition to the creation of permanent groups with mothers. Process mapping is being carried out and regular institutional support groups are being established with management.

It was also possible to notice that professionals felt insecure or partially safe when providing guidance for establishing and maintaining breastfeeding. In the baseline audit, almost half of the employees reported insecurity, despite the practice being carried out every day at the NU. We sought to resolve this difficulty through training for professionals from different categories, providing guidance on expressing milk at the bedside, establishing and implementing the flow of referrals for mothers to the HMB, systematizing and monitoring the kangaroo position in the neonatal unit, providing guidance on carrying out and recording skin-to-skin contact, and organizing and executing the reception flow for mothers of newborns and low birth weight babies in the service.

Another major barrier highlighted was the divergence of behavior and guidance from professionals and the discontinuity of work carried out within the sectors in which PTI/LBW breastfeeding occurs. With the aim of standardizing behaviors and improving this dialogue, strategies were used to develop and implement protocol and training of professionals.

Regarding absent mothers, there is still a lot of difficulty related to their bond with their children. Their difficulty in stimulating and expressing milk, as well as their resistance to placing the newborn in the kangaroo position and in skin-to-skin contact is noticeable. To this end, conversation circles and individual guidance were carried out aimed at changing behaviors, helping to maintain lactation, and ensuring that all mothers adopted the kangaroo mother care.

The critical point in developing the protocol was the team’s difficulty in defining when to start the transition from the oral tube to the mother’s breast. As a way of enabling more assertive behaviors, a specific seminar was promoted on the topic, entitled: “Breastfeeding preterm and low birth weight infants: transition from tube to oral diet”. Following the discussion with experts in the area, it was possible to notice greater dialogue between NU professionals, understanding that the breastfeeding binomial requires individual assessment and systematic reassessments, in addition to promoting team training in best evidence-based practices.

It was noted that the team was unaware of the Ten Steps to Successful Breastfeeding as guidelines for the breastfeeding process, and, to resolve this situation, training was scheduled on the Baby-Friendly Hospital Initiative.

The lack of a collection site (or place that operates 24 hours a day for extracting and storing milk) at the institution is also a factor that discourages breastfeeding. During nights, weekends and holidays, mothers who express milk at the bedside and do not use the total volume expressed need to discard the milk. Meetings with the management were held to provide adequate space and equipment for milk extraction and maintenance of milk production for these mothers.

Finally, the provision of milk formula as a supplement and the use of pacifiers occur at the NU for various reasons and without documents supporting their use. The team has discussed these issues and created documents supporting the use of formulas and pacifiers only in specific clinical conditions of the newborn.

The implementation strategy began in September 2022 and lasted 6 months, involving research leaders, heads of the maternal and child sectors, as well as NU employees and mothers. The proposed implementation schedule involved the following steps: analysis of the baseline audit; discussions with the working group; preparing and carrying out training with the team on the topic; development of an institutional breastfeeding protocol for preterm and low birth weight infants; and preparation and publication of institutional materials relating to the topic.

As a teaching strategy, theoretical-practical training/workshops were carried out, expanded to the entire multidisciplinary team in the institution’s maternal and child sectors, including: NU, rooming-in, pre-delivery, milk bank, pediatrics, and pediatric outpatient clinic. Sixteen meetings lasting two hours each were held, requiring employee participation in two meetings, a theoretical and a practical one, totaling four hours of course, for certification. We sought to make both meetings interactive and bring the reality experienced in each sector to be discussed in groups.

In the training, a dialogical presentation associated with different materials and interaction with the public took advantage of the participants’ prior knowledge for a deeper discussion about breastfeeding counseling and support practices, which are so important to keep mothers in the service and maintain their milk production through prolonged hospitalizations of newborns. The entire practice was based on JBI audit criteria^(19,20)^.

The workshops were held at four times a day, providing the opportunity for the largest possible number of professionals to participate. All occurred during working hours, ensuring more effective employee participation and promoting greater integration of the sectors. In contrast, the fact that they were face-to-face limited the number of participants, since participation was related to the demand for service and the possibility of leaving the sector during training hours. At the end of the training, the participation of 24% of the total number of employees in the maternal and child sectors and 56% of the total number of employees in the neonatal unit was recorded, with the participation of nurses, nursing technicians and assistants, doctors, physiotherapists, speech therapists, occupational therapists, psychologist, social worker, and administrative secretary.

The research leader remained available to answer questions and recruit employees who showed interest in participating in the breastfeeding support groups that are being established at the hospital.

**Figure 1:**
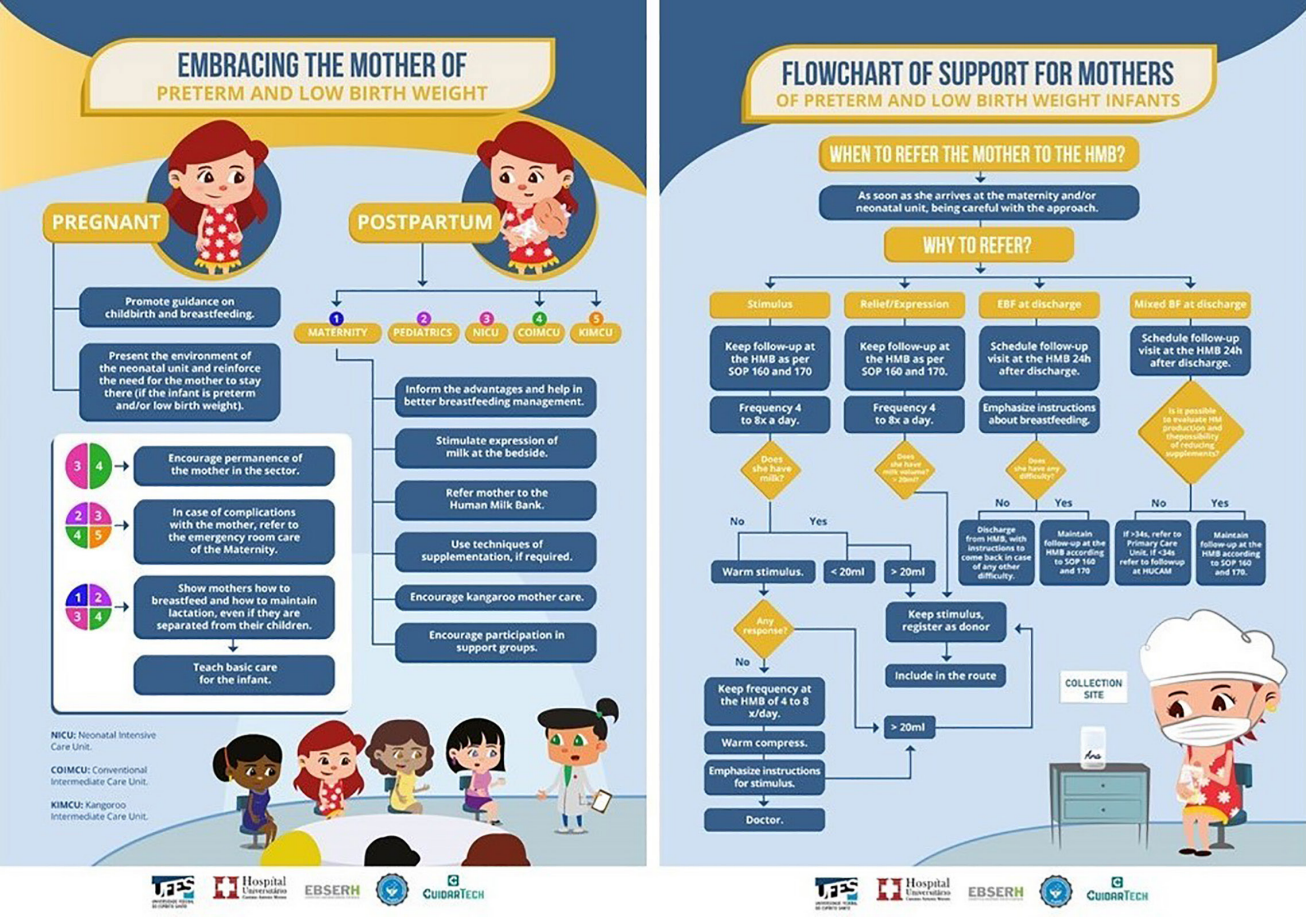
Protocol support materials with descriptions of routines and care flows.

As a result of the strategies implemented, the first institutional breastfeeding protocol for preterm and low birth weight infants was developed and approved, with the establishment of routines and standardization of behavior. Intersectoral training was also carried out with the multidisciplinary team, supporting documents for the protocol were prepared and approved (such as SOPs), and routine guiding documents were established, such as the process mapping and flowchart presented in Figure 1.

### Follow-up Audits

**Figure 2:**
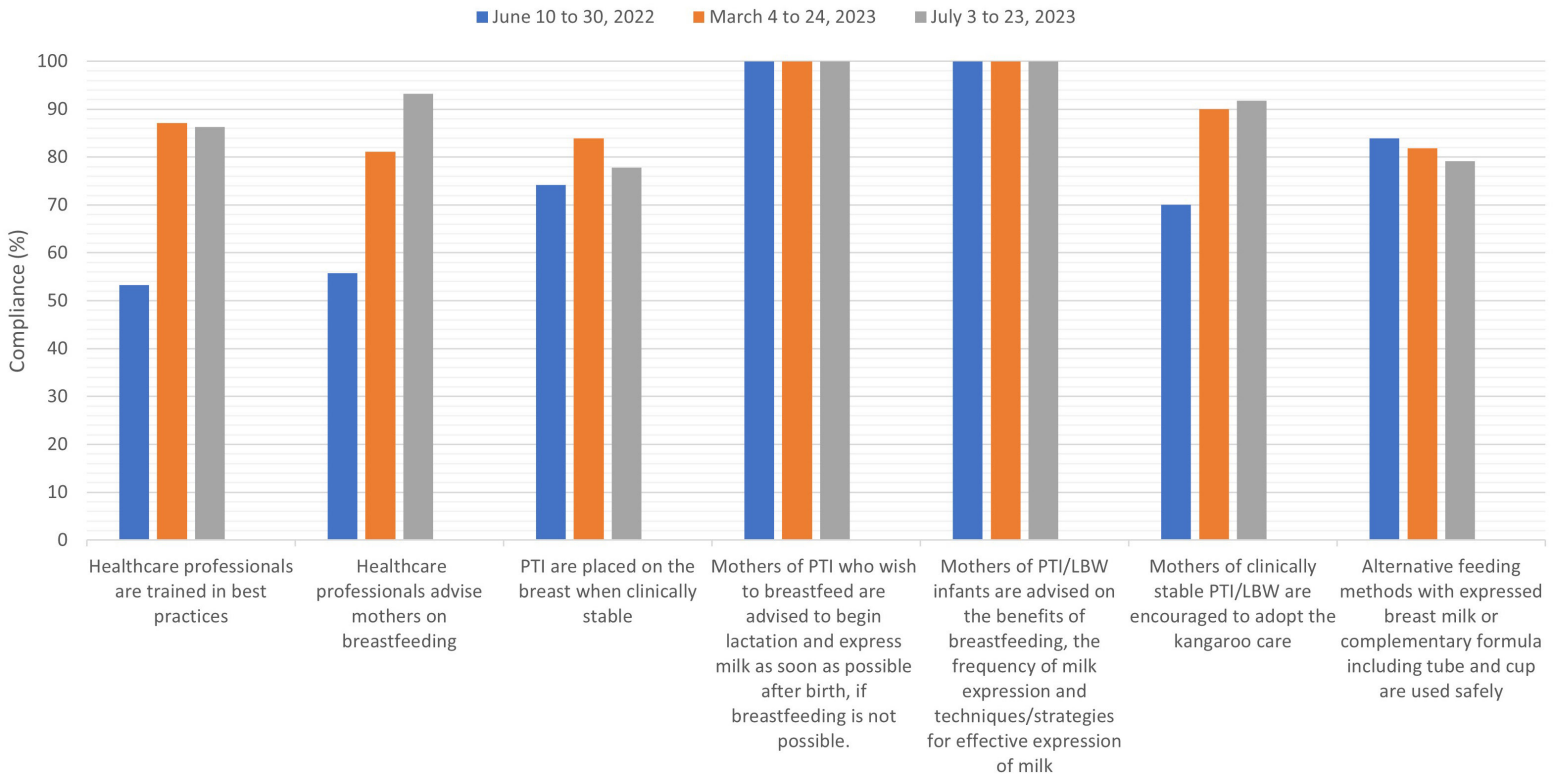
Description of compliance (%) with best practices for audit criteria for breastfeeding of preterm and low birth weight infants in the baseline audit between June 10th and 30th, 2022 and in follow-up audits between March 4th and 24th and July 3 and 23, 2023.

Note: PTI – Preterm infant; LBW – Low birth weight.

Data from the first follow-up audit was collected by the project leader for 20 days, one month after the end of the training, from March 4 to 24, 2023. Sixty-two employees and 10 mothers participated in this stage. This audit cycle proved that the results were satisfactory and that all criteria achieved compliance greater than 80% - with two of them maintaining 100% compliance, as seen in Figure 2 - and criteria 1 and 2 had the largest increases in rates after implementation. The aim was to carry out the first follow-up audit one month after the training and the second audit at least four months later, as the methodology used classifies this last period as necessary for the sedimentation of new practices^(15,19)^. It should be noted that data collection for this research was entirely guided by the JBI method.

In an individual analysis of each criterion, it was possible to notice that there was a significant increase in the compliance rates of four (1, 2, 3 and 6) of the seven criteria audited, and all of them became compliant (> 80%). Criteria 1 and 2, which had the lowest compliance (53.3% and 55.8%), increased to 87.1% and 81.1%, respectively. Criterion 3 increased from 74.1% to 83.9% in the follow-up audit. Criterion 6, which analyzed whether clinically stable PTI/LBW infants were placed in the kangaroo position, increased from 70 to 90%. It is interesting to point out that the only mother who stated that she had not performed the kangaroo care in the follow-up audit had her child in a serious condition, using devices and vasoactive drugs since birth, with limited possibility of performing the kangaroo position. This mother was present from the beginning of hospitalization and expressed milk at the bedside at all diet times.

Criteria 4 and 5 maintained 100% compliance in baseline and follow-up audits. Criterion 7 was the only one that showed a reduction in compliance from 83.88 to 81.88%. This reduction may be associated with the fact that employees, after training, respond more legitimately about their ability to perform the techniques.

Figure 2 also presents the second follow-up audit, which took place between July 3 and 23, 2023 and was attended by 73 employees and 12 mothers. The cycle of this audit showed that the indexes remained better than in the baseline audit; however, there was a drop in the values of three criteria when compared to the first follow-up audit. In criterion 1 there was a reduction in compliance from 87.1 to 86.3%, as well as in criteria 3 and 7, in which the reduction in compliance occurred from 83.9 to 77.8% and 81.88 to 79.1%, respectively.

Criteria 2 and 6 increased from 81.1 to 93.2% and from 90 to 91.8%, characterizing the emphasis given by health professionals when guiding mothers and family members regarding breastfeeding and performing the kangaroo care.

## DISCUSSION

The organization and implementation of the protocol allowed the standardization of behaviors among health professionals and organization of the work process. The reduction in variation in the care provided directly reflects the improvement in the quality of care. A more uniform service can be made possible by access to knowledge about best practices, synthesis of evidence, leadership training, team education and training, implementation of new practices, and evaluation of new behaviors^(14,15,18,21)^.

Initiating and maintaining breastfeeding in PTI/LBW infants is complex. The main challenges to breastfeeding and providing human milk include lack of parental knowledge about the benefits of human milk, difficulties with lactation and milk expression, maternal stress and fatigue, nature of the neonatal intensive care environment, delay in beginning of expression, and the physical separation of babies from their mothers^(5,22,23)^.

The formation of well-trained teams prepared to deal with this process, most of the time long, is essential to achieve satisfactory results close to discharge. The combination of scientific knowledge, care environment, and family-centered practice are effective strategies to promote an increase in the volume of breast milk and improved breastfeeding for mothers separated from their preterm infants admitted to a neonatal intensive care unit^(2,3,6,9,11)^. Changes in hospital practices in accordance with the Ten Steps to Successful Breastfeeding and the training of professionals increase breastfeeding prevalence^(7,22–24)^.

It is possible to find an increasing number of works evaluating the impact of implementing clinical practice guideline programs on breastfeeding, as well as their quantitative and qualitative indicators, with the aim of adapting clinical practice to new knowledge, reducing the cost of changes and improving the likelihood of success^(23–26)^. Examples of this are the implementation of the practices proposed by BFHI-Neo (BFHI for Neonatal Units) in a hospital in southeastern Brazil, which showed a significant change in breastfeeding at discharge and the empowerment of families to care for their preterm children, allowing for more support measures to be achieved and, consequently, for the improvement in the prevalence of breastfeeding in preterm infants^(23)^ and the increase in exclusive breastfeeding rates at hospital discharge of newborns admitted to neonatal units, after the promotion of early and frequent expression of breast milk among mothers of preterm babies in a hospital in Shanghai, China^(27)^.

PTI/LBW infants present unique needs and challenges and require highly specialized and personalized strategies when it comes to optimal nutrition. It is worth noting that they require specific breastfeeding policy for neonatal intensive care and determine that health professionals have knowledge and skills on lactation and breastfeeding support, including the provision of specific prenatal information and care, characterized by the facilitation of early, continuous and prolonged skin-to-skin contact (kangaroo mother care), early initiation of milk expression and breastfeeding and mothers’ access to breastfeeding support throughout the baby’s hospitalization^(8,11,23,26,27,28,29)^.

Providing human milk is the primary goal for these babies’ health, and supporting practices are crucial for this to happen. The behavior and stability of the newborn (NB) should guide the initiation of oral feeding attempts, as gestational age and weight are not capable of encompassing the normal variability observed in development or the impact of comorbidities presented by the NB throughout the process. Oral feeding plans should be individualized based on the NB’s behaviors and performance, as well as their overall progress^(3,4,6–8)^.

When the infant has an adequate sucking pattern and clinical stability, the tube-to-oral transition happens earlier, reducing tube feeding time. Avoidance of use of bottles increases the prevalence of exclusive breastfeeding at discharge and improves rates of exclusive breastfeeding up to six months after discharge^(3,18)^.

In general, the need for supplementation in PTI/LBW infants is a routine practice until nutritive suction is safely established. Best evidence-based practices bring alternatives so that the completion time is reduced in the reality of NUs, through techniques such as: skin-to-skin contact; early and frequent expression of milk; delay reduction in starting breastfeeding; assurance of good positioning and latching of the newborn, and permanence of the mother 24 hours with her child^(2,7,8,11,12,22)^. In this scenario, it is extremely important for the neonatal unit to have the kangaroo mother care implemented and all employees trained to facilitate breastfeeding practices.

Different interventions can be applied in implementation research to achieve quality improvement related to breastfeeding support and maintenance practices^(13,25)^. A study focused on quality improvement initiatives for hospitalized newborns more frequently found the use of in-service training, distribution of reference materials, strengthening of facility infrastructure, and feedback as interventions to achieve quality improvement^(30)^.

Implementing evidence into practice is not an easy or automatic process, as it involves changing behavior towards a new mentality and a new culture at a personal and organizational level. Scientific evidence can be put into practice through implementation science, with the application of systematic research and the evaluation of the results obtained. The science of implementation provides tools for implementing the best evidence and its use through intervention projects, models and protocols that aim to reduce inconsistencies and leverage health results^(14,15,27)^.

Integrated work among multidisciplinary teams with standardization of care and the use of a protocol with clear roles and responsibilities, with an organized flow of care and frequent training, are fundamental factors in reducing individual differences that directly affect quality and allow implementing best evidence into clinical practice^(21,31)^.

As limitations of the study, follow-up audits took place at one and five months respectively, after theoretical-practical training. This deadline was directly related to the need to complete the research, linked to a professional master’s degree in nursing.

Furthermore, this study did not consider patient preferences, as the protocol was not evaluated by users, even though it used the best available evidence, professionals’ experience, and existing resources. New follow-up audits must be carried out to ensure the sustainability of the new practices implemented.

## CONCLUSION

This research developed and implemented the first institutional breastfeeding assistance protocol for preterm and low birth weight infants and improved the evidence-based practice of the multidisciplinary team, through the implementation of multifaceted strategies aimed at the identified barriers.

The main facilitating tools were the establishment of the working group, intersectoral training for the multidisciplinary team, provision of constructed documents related to the topic, and listening to mothers and health professionals. The protocol and part of the products are already implemented and generating positive impacts on the institution. The benefit of the training being in-person is highlighted, as there was great interaction among the participants and also rich moments of exchanging experiences, especially between different sectors.

The documents produced improved communication and the organization of the workflow in the service, facilitating dialogue among the sectors and professionals who provide assistance to the binomial. The difficulties could be discussed more closely with the joint construction of actions that served the mother and the newborn in different sectors.

The multidisciplinary team was trained according to best evidence-based practices; however, the number of professionals was still low considering the total number of employees in these sectors. Furthermore, professionals’ personal beliefs and experiences continue to be a strong challenge related to the guidance that should be offered to mothers and family members.

It is worth noting that after the training, health professionals were more engaged in guiding and assisting mothers in breastfeeding, improving referral and attendance rates at the Human Milk Bank, as well as exercising a more uniform language within the team, as seen during multidisciplinary visits and subsequent follow-up audits. At this time, guiding documents for the team were also established at the institution, such as SOPs, flowcharts, and process mapping. The project was successful in increasing knowledge in this area and providing guidance to sustain change.

This research has the potential to be applied at a regional and national level, as it describes an innovative method systematically, with the steps and resources required to implement the best scientific evidence. The study influenced changes in practice, but maintaining evidence-based care recommended in the protocol requires ongoing education of the neonatal team and other sectors involved to assist with adherence and better results.
